# Employing skin self-examination and fear of cancer recurrence management in early-stage melanoma follow-up: evaluation of the MELACARE intervention in a randomised controlled trial

**DOI:** 10.1007/s11764-025-01841-1

**Published:** 2025-06-12

**Authors:** Sara Mølgaard Hansen, Christoffer Johansen, Nadine A. Kasparian, Mia Klinten Grand, Pernille Envold Bidstrup, Lisbet Rosenkrantz Hölmich

**Affiliations:** 1 Department of Plastic Surgery, Copenhagen University Hospital, Herlev and Gentofte Hospital, Herlev, Denmark; 2 Department of Oncology, Cancer Survivorship and Late Effects Research Center (CASTLE), Copenhagen University Hospital, Rigshospitalet, Copenhagen, Denmark; 3 Heart and Mind Wellbeing Center, Heart Institute and Division of Behavioral Medicine and Clinical Psychology, Department of Pediatrics, Cincinnati Children’s Hospital Medical Centerand, University of Cincinnati College of Medicine, Cincinnati, OH USA; 4 Statistics and Data Analysis, Danish Cancer Institute, Copenhagen, Denmark; 5 Psychological Aspects of Cancer, Danish Cancer Institute, Copenhagen, Denmark; 6 Institute of Psychology, University of Copenhagen, Copenhagen, Denmark; 7 Department of Clinical Medicine, University of Copenhagen, Copenhagen, Denmark

**Keywords:** Melanoma, Fear of cancer recurrence, Skin self-examination, Nurse-led follow-up, Randomised controlled trial

## Abstract

**Purpose:**

The MELACARE intervention aimed to evaluate a nurse-led follow-up program incorporating skin self-examination (SSE) education and psychosocial support to address fear of cancer recurrence (FCR) in early-stage melanoma survivors. This study assessed the MELACARE intervention’s impact on FCR, psychological well-being, SSE performance, and healthcare usage compared to standard physician-led follow-up.

**Methods:**

A two-group randomised controlled trial was conducted at Herlev and Gentofte Hospital, Denmark. Participants included 153 patients with surgically treated melanoma (stages IA–IIA). Patients were randomised to either the MELACARE intervention (*n* = 78) or a control group provided treatment as usual (*n* = 75). The intervention involved nurse-led sessions focusing on SSE techniques and metacognitive strategies. Outcomes included FCR (primary), distress, anxiety, depression, health-related quality of life (HRQoL), patient activation, and SSE frequency and confidence (secondary) at 6 months. Trial registration: ClinicalTrials.gov (NCT05253872).

**Results:**

At 6 months, the mean of the primary outcome FCR was lower in the intervention compared to the control groups, but the difference was not statistically significant (− 0.86 [− 3.34;1.62]). Intervention patients reported higher HRQoL (18% [3;32]) and patient activation (0.43 [0.15;0.71]) as the only significant secondary outcomes. Confidence in SSE was higher in the intervention group, with most performing SSE at recommended intervals.

**Conclusions:**

The MELACARE intervention may improve HRQoL and patient activation but did not reduce FCR. High fidelity of delivery and patient adherence highlight its potential utility.

**Implications for Cancer Survivors:**

The MELACARE approach empowers melanoma survivors through structured SSE education and psychosocial support. Future analyses will investigate long-term safety and efficacy.

**Supplementary Information:**

The online version contains supplementary material available at https://doi.org/10.1007/s11764-025-01841-1.

## Introduction

The incidence of cutaneous melanoma, as well as the number of melanoma survivors, continues to rise, resulting in a growing population of patients requiring follow-up after melanoma surgery [1, 2]. Post-surgical follow-up care includes monitoring for new and recurrent melanoma through regular physician-performed skin exams [3, 4], which are resource-intensive for the healthcare system [5]. The cost-effectiveness of the current follow-up regarding recurrences detected at scheduled appointments compared to recurrences detected by patients between appointments is unknown [6–9]. With almost three-fourths of recurrences being discovered by patients between scheduled follow-up visits [10], depending on the stage and use of imaging, skin self-examination (SSE) is an important source of detection.

Different SSE education modalities, e.g. in-person, workbooks, tablets, and apps, have demonstrated a positive impact on SSE frequency, thoroughness, and confidence [11–18]. However, all previous interventions have been evaluated as adjuncts to regular physician-performed skin exams [11–18]. Clinical experience suggests that many patients feel reassured after skin exams conducted by their physician, while some express reluctance to perform SSE [19]. This hesitation may be attributed to the perceived difficulty of SSE and a concern that it could induce or increase fear of cancer recurrence (FCR); however, this issue has not been previously evaluated.

Although the risk of recurrences is relatively low for early-stage melanoma [20], up to 75% report clinically significant levels of FCR [19, 21, 22]. FCR is the most frequently reported unmet need in melanoma survivors [23], is closely associated with anxiety and depression, and may result in reduced health-related quality of life (HRQoL) [24] as well as increased healthcare use [25, 26]. Four previous studies have established the effect of psychosocial support on lowering FCR in patients treated for melanoma. An RCT (the Melanoma Care Program) with 164 stage 0–II melanoma patients showed that a telephone-based psychoeducational program significantly reduced FCR at 6 months compared to usual care [27]. The ConquerFear program, tested in an RCT with 222 cancer survivors, including four melanoma survivors, also reduced FCR up to 6 months post-intervention using metacognitive and self-regulation therapy [28]. Two RCTs tested online interventions, including a mindfulness program for stage IIC–III melanoma patients [29] and a cognitive behavioural therapy-based program for early-stage cancer (only four melanoma survivors included) [30], both showing modest FCR reductions.

Using an RCT design, we examined whether the MELACARE intervention with SSE education as recurrence surveillance in combination with psychosocial support could reduce FCR, improve psychosocial well-being, and increase SSE performance without compromising the detection of new recurrences, compared to regular physician-led follow-up at 6 months’ follow-up. We theorised that psychosocial support may prevent or alleviate FCR, and SSE may detect recurrence.

## Methods

### Study design

**Fig. 1:**
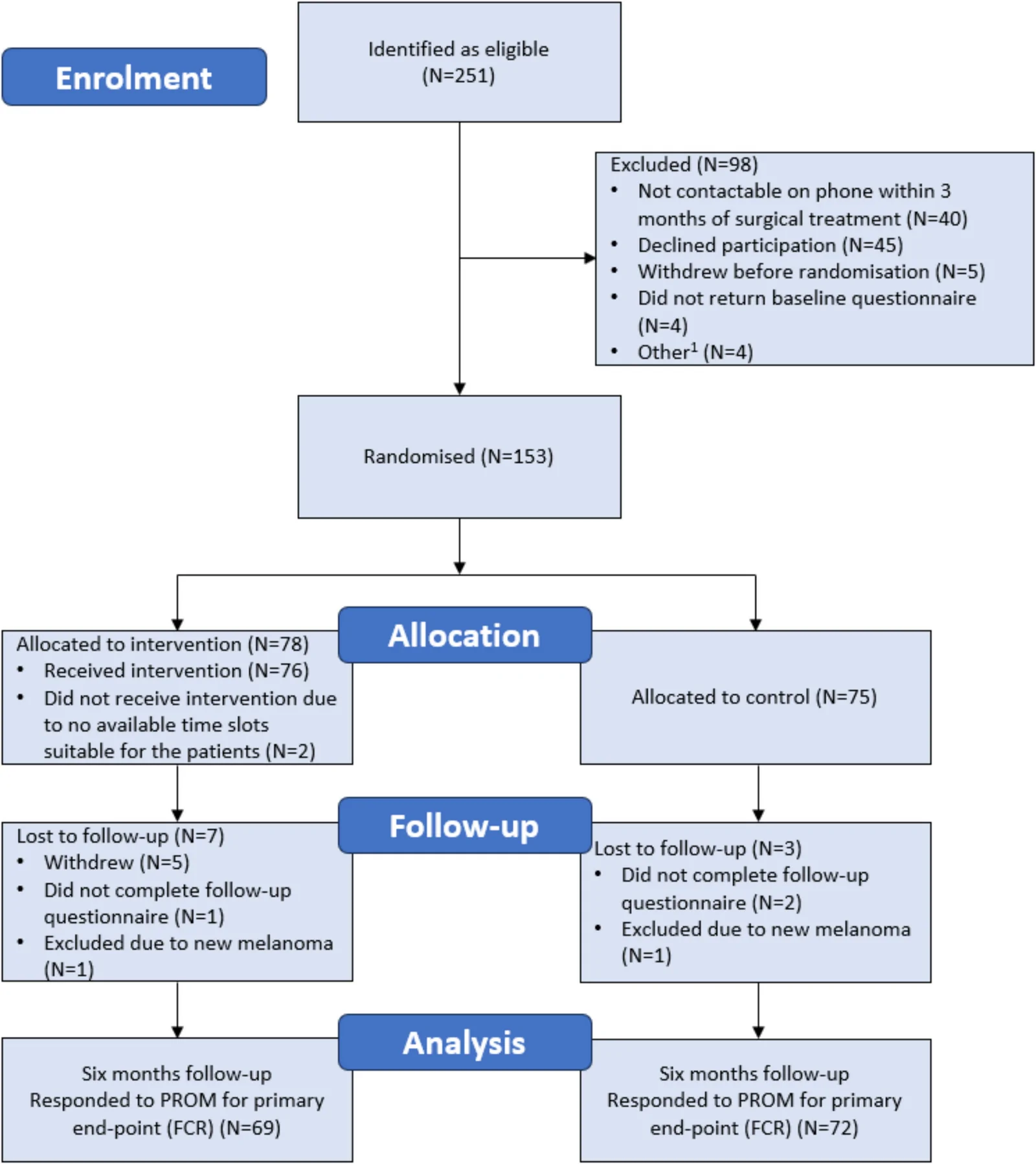
CONSORT diagram. FCR, fear of cancer recurrence; PROM, patient-reported outcome measure. ^1^Not permanently living in Denmark or moved abroad

MELACARE was tested using a two-group RCT in which patients were allocated equally to the intervention or usual care (Fig. 1); this has previously been described [31]. The study was conducted at the Department of Plastic Surgery, Copenhagen University Hospital, Herlev and Gentofte, Denmark. We followed CONSORT [32] and TIDieR for reporting [33].

### Study population and recruitment

Patients were eligible for inclusion if they had been surgically treated for melanoma stages IA–IIA within 3 months of recruitment, had no prior history of other cases of melanomas or other cancers excluding non-melanoma skin cancer, were aged 18 years or more, and had sufficient proficiency in Danish. Exclusion criteria included a high risk of a new melanoma (i.e. presence of dysplastic nevus syndrome or a first-degree relative with melanoma), comorbidity that made SSE teaching impossible (e.g. physical or mental disabilities), and no sentinel node detected in the case of lymphoscintigraphy. Patients were screened for eligibility by the principal investigator (SMH, medical doctor) and were consecutively recruited by telephone by the principal investigator, a project nurse, or a medical student between March 9, 2021, and September 28, 2023.

### Random assignment

Random assignment was computer-generated and performed using a closed site (www.melanoma.dk) in blocks of eight patients. Participants were randomised 1:1 to the intervention or control group and stratified according to clinical stage (IA vs. IB vs. IIA). Allocation was blinded to the participants until random assignment. After random assignment, blinding of the allocation to participants, the principal investigator, physicians, and project and melanoma nurses was impossible.

### Control group

**Fig. 2:**
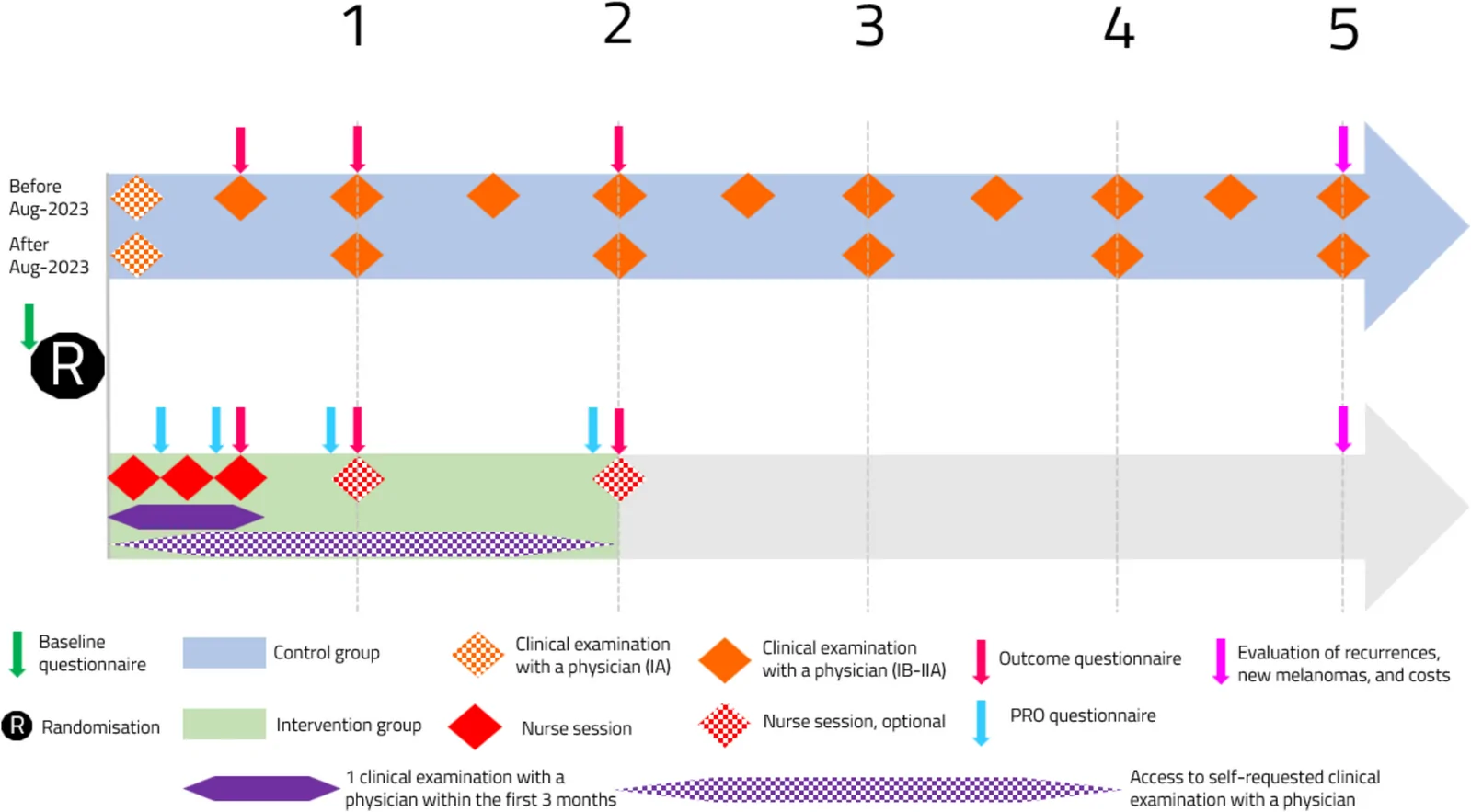
Study schedule of trial activities in the intervention and control groups. Aug, August; PRO, patient-reported outcomes

Patients in the control group received treatment as usual with follow-up according to their clinical stage (Fig. 2). Follow-up consists of regular physician-performed skin and lymph node exams. Patients with stage IA have one follow-up appointment at 3 months, whereafter they are discharged. Patients with stages IB–IIA have one follow-up appointment every 12 months for 5 years.

### Intervention

The intervention aimed to educate patients in SSE for recurrence detection and to provide psychosocial support to reduce FCR through nurse-led follow-up. The following are the components of the intervention follow-up:

1. Three to five nurse-led follow-up in-person individual sessions within the first 2 years of randomisation (Fig. 2, Online Resource 1). The main focus of the sessions was to provide information on and practice SSE, provide information on which symptoms would require the patient to contact the hospital department, and educate patients on coping with FCR using the metacognitive strategies detached mindfulness (DM) and worry postponement (WP) [31], both of which were part of previous successful intervention studies [27, 28]. Nurses delivering the intervention received a 2-day training program from a medical doctor and two psychologists (Online Resource 2). The nurse sessions were supported by:
2. The “Melanoma – Questions and Answers” booklet [34] with information on melanoma, SSE, and coping strategies.
3. Patient-reported outcome (PRO) assessment using the melanoma-specific HRQoL questionnaire “Functional Assessment of Cancer Therapy – Melanoma” (FACT-M) [35, 36]. The items were discussed at the sessions, and action was taken according to predefined guidelines (Online Resource 3) if any items exceeded predefined thresholds.
4. One scheduled appointment with a physician (plastic surgeon) was conducted to ensure the participants’ eligibility to perform SSE (Fig. 2). Hereafter, no regular appointments with a physician were scheduled, but self-requested appointments were available throughout the study period [31].

### Data collection

Baseline socio-demographic data, including marital status, education, number of children, and household income, were collected electronically through a questionnaire using the Research Electronic Data Capture (Vanderbilt University, Nashville, TN). Baseline clinical data, including age, gender, and melanoma stage, were collected through the medical records. PRO data on FCR, anxiety, depression, distress, HRQoL, workability, and patient activation were collected through validated questionnaires at baseline and after 6, 12, and 24 months of follow-up. Information on SSE frequency and confidence, satisfaction with the follow-up program, and use of DM, WP, and booklet were collected through a study-specific questionnaire at 6, 12, and 24 months of follow-up (Online Resource 4). Clinical data, including recurrences, new primary melanomas, additional unplanned physician consultations, excisions, or scans, were collected through the medical records.

All in-person nurse sessions were audio recorded (with patient permission) to assess the fidelity of the intervention delivery, and two independent raters assessed whether the nurses delivered the expected intervention components to the patients at Sessions 1 and 2. Information on session duration, FACT-M completion, and actions taken due to FACT-M responses was registered by the nurse after each session.

### Outcomes

#### Primary outcome

The primary outcome was FCR, measured using the Danish version of the Concerns About Recurrence Questionnaire 4 (CARQ-4) [36, 37] at 6 months follow-up. CARQ-4 is a validated four-item scale with a total score ranging from 0 to 40; higher scores indicate greater FCR levels, and scores above 11 indicate FCR warranting clinical assessment [37]. No minimal clinically important difference (MCID) has been described for CARQ-4.

### Secondary outcomes

Secondary outcomes included several PROs: anxiety, depression, distress, HRQoL, workability, patient activation, number of SSEs, use of DM and WP, use of the booklet, and satisfaction with the follow-up program. Anxiety was measured using Generalised Anxiety Disorder-7 (GAD-7), which has seven items, scores ranging from 0 to 21, scores ≥ 10 indicating significant anxiety, and higher scores indicating more profound anxiety [38, 39]. The Patient Health Questionnaire-9 (PHQ-9) was used to measure symptoms of depression; it has nine items, and scores range from 0 to 27, where higher scores indicate a higher risk of having depression, and a cut-off of ≥ 10 indicates moderate to severe depression [40, 41]. Distress was assessed using the distress thermometer (DT), a single-item tool where patients rate their distress on a scale from 0 (no distress) to 10 (extreme distress), and we used the cut-off of 3 or above to identify patients with significant distress [42, 43]. We used the EuroQol 5-Dimensions 3-Levels questionnaire (EQ-5D-3L) to evaluate HRQoL [44]. It evaluates five dimensions of health, each with three severity levels, and includes a visual analogue scale for overall health rating. Raw scores are transformed into a utility score that ranges from 0 to 1, with scores closer to one indicating better health status. Workability was evaluated using the Work Ability Index (WAI), a 7-item scale ranging from 7 to 49; higher scores indicate better workability, and scores of 27 or under are considered poor workability [45, 46]. The Patient Activation Measure (PAM) was used to assess patient activation. PAM includes 13 items, and scores are converted into levels that range from low activation (Level 1) to high activation (Level 4) [47].

Secondary outcomes also included recurrences, new melanomas, additional unplanned physician consultations, excisions, and scans, as well as intervention fidelity measured as adherence to the protocol and delivery, session duration, FACT-M completion rate, and actions taken due to FACT-M responses (e.g. referral to physiotherapy).

### Sample size and power calculation

We calculated the sample size a priori and expected effect size of Cohen’s *d* of at least 0.3 on the primary outcome, as found in a similar study at 6-month follow-up [27]. With an expected attrition rate of 10% and a power of 79% to detect a small effect size of Cohen’s *d* = 0.3, 340 participants were needed (170 per group).

### Statistical analyses

The primary outcome was FCR after 6 months of follow-up. The intervention effect, comparing the mean outcome of FCR after 6 months under the intervention regime to the control regime, was estimated using targeted maximum likelihood estimation (TMLE) [48]. TMLE was also used to estimate the intervention effect for the secondary outcomes of distress, anxiety, depression, HRQoL (dichotomised as 1:HRQoL = 1 and 0:HRQoL < 1), patient activation, and workability. TMLE was furthermore used in the subgroup analyses assessing the effect on FCR within the subgroups of FCR at baseline (> 11 or ≤ 11), age (≥ 60 or < 60), sex, and civil status. Since HRQoL was considered a binary outcome, the effect was reported as the risk difference between the intervention and control regimes. In analyses of the other outcomes, the effect was reported as the mean difference.

TMLE was used instead of the protocol’s proposal of linear mixed models [31] to obtain a marginal intervention effect estimate, adjust for imbalance in baseline covariates, relax normality assumptions, and provide more accurate confidence intervals and *p*-values. Details about the TMLE analyses can be found in the appendix (Online Resource 5). Additional analyses, including those from the protocol paper, can be found in the appendix (Online Resource 6).

SSE-related outcomes; satisfaction with the follow-up program; the use of DM, WP, and the booklet; and the number of extra physician consultations, unplanned scans, and extra excisions were calculated as percentages for each group. Overall fidelity was calculated as the percentage of intervention components delivered relative to the total planned components. Interrater fidelity was measured as the proportion of agreement between the two raters.

All tests were two-sided with a significance level of 0.05. The data analyses were performed using the R package tmle [49] and R version 4.3.3.

## Results

A total of 153/211 eligible patients enrolled in the study (73%) and were randomised to the intervention (*n* = 78) or control group (*n* = 75) (Fig. 1). In the intervention group, five participants withdrew (one before the first nurse session, three after one nurse session, and one after two nurse sessions), and one was excluded due to a new melanoma. All that withdrew did so because they wanted regular physician-performed skin exams instead. In the control group, no one withdrew, and one was excluded due to a new melanoma (Fig. 1).

### Patient characteristics

Patients in the intervention group were younger (60 vs 65 years), more were still working (58 vs 48%), and more had an annual family income of 54.000 EUR or more (78 vs 68%). All other patient characteristics were equally distributed (Table 1). At baseline, 46% of the patients had clinical FCR levels (Table 1). No patients had recurrence in either group within the first 6 months of follow-up.

**Table 1:** Patient characteristics among 153 randomised patients at baseline according to group status

Characteristic	Control (*n* = 75)	Intervention (*n* = 78)	Total (*n* = 153)
Mean age (SD)	65 (14)	60 (12)	62 (13)
Sex			
Men	38 (51)	38 (49)	76 (50)
Women	37 (49)	40 (51)	77 (50)
Education			
Primary education, secondary education or academy profession degree or similar	32 (43)	32 (41)	64 (42)
Bachelor’s degree or higher	43 (57)	46 (59)	89 (58)
Employment status			
Working	36 (48)	45 (58)	81 (53)
Retired	37 (49)	32 (41)	69 (45)
Other	2 (3)	1 (1)	3 (2)
Civil status			
Married/cohabitating	58 (77)	56 (72)	114 (74)
Separated/divorced/never married/widowed	17 (23)	22 (28)	39 (26)
Have children			
Yes	68 (91)	69 (89)	137 (89)
No	7 (9)	9 (11)	16 (11)
Median no. of children (IQR)	2 [2, 3]	2 [2, 3]	2 [2, 3]
Yearly family income (before tax EUR)			
53.999 or under	22 (32)	16 (23)	38 (27)
54.000 or more	46 (68)	55 (77)	101 (73)
Did not want to answer	7	7	14
Stage^1^			
IA	50 (67)	54 (69)	104 (68)
IB	23 (30)	22 (28)	45 (29)
IIA	2 (3)	2 (3)	4 (3)
Fear of cancer recurrence			
< 12	38 (51)	44 (56)	82 (54)
≥ 12	37 (49)	34 (44)	71 (46)

*SD* standard deviation, *IQR* interquartile range, *EUR* Euro

^1^Stages IA, IB, and IIA refer to the American Joint Committee on Cancer, 8 th edition melanoma classification

### Intervention effect

#### Psychosocial well-being

At 6 months, mean FCR under the intervention regime was non-significantly lower than under the control regime (mean difference of − 0.86 95% CI [− 3.34;1.62]). The intervention also non-significantly lowered the secondary outcomes of distress, anxiety, and depression and non-significantly increased workability compared to patients in the control group (Table 2). The intervention significantly increased the secondary outcomes HRQoL (risk difference of 18% 95% CI [3;32]) and patient activation (mean difference of 0.43 95% CI [0.15;0.71]) among the intervention group compared to the control group (Table 2). Subgroup analyses showed no significant impact on FCR (Online Resource 7). Linear mixed model analyses, as proposed in the protocol [31], showed a similar result of the intervention effect as the TMLE analyses (Online Resource 6).

**Table 2:** Summary of the distribution at baseline and 6-month follow-up, along with the intervention effect on fear of cancer recurrence, distress, anxiety, depression, workability, patient activation, and health-related quality of life

Outcome	Baseline		Six months		Between-group difference	
	Control (*n* = 75)	Intervention (*n* = 78)	Control (*n* = 72)	Intervention (*n* = 69)	Estimate (95% CI)	*p*-value
	Mean (SD)		Mean (SD)		Mean difference^1^	
Fear of cancer recurrence	12.7 (9)	12 (9.9)	10.1 (9.2)	8.6 (9)	− 0.86 (− 3.34;1.62)	0.49
Distress	2.5 (2.9)	2.1 (2.5)	1.6 (2.2)	1.1 (2)	− 0.3 (− 0.91;0.31)	0.33
Anxiety	3.1 (3.6)	3 (3.8)	2.7 (3.8)	2 (3.7)	− 0.53 (− 1.45;0.4)	0.27
Depression	2.9 (3.8)	3 (3.8)	2.6 (3.9)	2.1 (3.8)	− 0.68 (− 1.72;0.37)	0.2
Workability	40.2 (5)	38.9 (8.7)	40.9 (5.5)	41.9 (8.1)	1.35 (− 0.86;3.56)	0.23
Patient activation	2.9 (0.9)	2.8 (0.9)	2.9 (0.8)	3.3 (0.9)	0.43 (0.15;0.71)	0.003
	*N* (%)		*N* (%)		Risk difference (%)	
Health status^2^ < 1	34 (45)	41 (53)	35 (50)	19 (28)	18 (3;32)	0.02
Health status^2^ = 1	41 (56)	37 (47)	35 (50)	49 (72)		

*SD* standard deviation, *CI* confidence interval

^1^Targeted maximum likelihood estimation (TMLE) was used to estimate the intervention effect at 6 months’ follow-up

^2^Health status is related to the health-related quality of life measure EQ-5D-3L, where a health status of 1 indicates “perfect health”

#### Self-examination skills

**Fig. 3:**
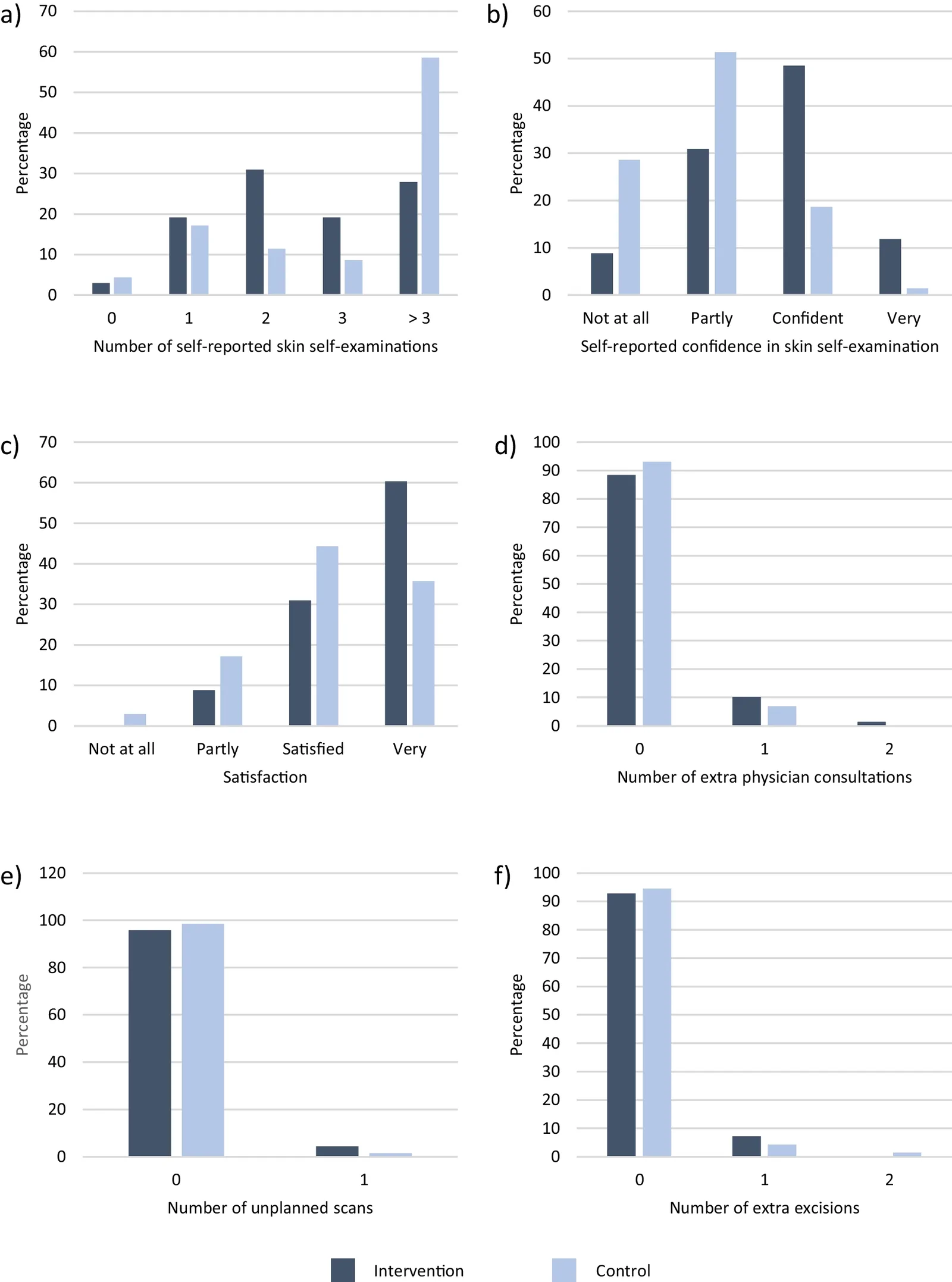
The proportion of self-reported **a** frequency of skin self-examinations, **b** skin self-examination confidence, **c** satisfaction, and **d**–**f** use of healthcare services in the intervention group and control group at 6 months of follow-up (*N* = 141)

Most patients in the intervention group (69%) performed SSE one to three times during the 6-month follow-up period (they were instructed to perform SSE approximately every third month). In contrast, most patients in the control group (59%) performed SSE more than three times (Fig. 3). Patients in the intervention group reported being more confident performing SSE and satisfied with the follow-up than patients in the control group (Fig. 3). Only minor differences in the proportion of healthcare use were seen between the two groups (Fig. 3).

### Delivery, fidelity, and acceptability of the intervention

The average duration of sessions decreased from 46 to 34 and 21 min across Sessions 1–3. All patients completed FACT-M before Session 1 and 2, and 91% completed it before Session 3. Advice to see the GP, request a consultation at the outpatient clinic, or help and support provided through the sessions were the most frequent actions due to the FACT-M replies (Online Resource 8). Intervention fidelity was 91%, as assessed by two independent raters, with an interrater fidelity of 99%.

Fifty percent of the patients in the intervention group did not use the DM and WP coping strategy tools, and around 40% found them “not at all useful”. Most patients agreed or completely agreed that they have learned how often to perform SSE (100%) and how to perform SSE (99%), cope with their melanoma diagnosis (94%), and use DM and WP coping strategies (77%) (Online Resource 9).

## Discussion

We found that MELACARE, a new follow-up method for patients surgically treated for early-stage melanoma, including SSE education and psychosocial support delivered by specially trained nurses, did not statistically significantly lower FCR, distress, anxiety, depression, or improve workability at 6 months’ follow-up. Still, we did find indications of a positive effect of the intervention on HRQoL and patient activation. Inclusion had to be terminated prematurely due to a critical lack of nurses. The calculated sample size was thus by far obtained, which is likely to have influenced the power of our study.

### Fear of cancer recurrence and psychological symptoms

As we speculated that SSE might increase FCR, it was important to ensure this would be mitigated through psychosocial support specifically targeting FCR. For all the psychological symptoms (FCR, distress, anxiety, and depression), we did see lower symptoms at 6 months in the intervention group compared to the control group; however, the difference was not statistically significant. Unfortunately, we are not able to evaluate whether the change in FCR is clinically relevant, as no measure of minimal clinically important difference (MCID) has been established for the CARQ-4. Previous RCTs, including the Melanoma Care program, ConquerFear, and two online interventions, found significant reductions in FCR, but they had sufficient sample sizes (between 69 and 222 patients included in those RCTs) [27–30]. A large part of the patients did not use the coping strategies or found them useful. This might be because 56% did not have clinically significant FCR levels at baseline, suggesting that learning metacognitive tools may not be relevant to all patients with early-stage melanoma. Future studies could consider screening for FCR and testing the MELACARE intervention among patients with elevated FCR.

While we chose a standard superiority trial design to evaluate the potential benefits of the MELACARE intervention, we acknowledge that a non-inferiority design may have been equally, if not more, appropriate given the broader healthcare implications of demonstrating that less intensive, patient-led follow-up with SSE and psychosocial support does not lead to worse outcomes. Showing non-inferiority in FCR outcomes compared to physician-led follow-up could in itself be a valuable contribution, particularly in the context of healthcare resource optimisation and patient empowerment.

### Skin self-examination

A larger proportion of patients in the control group performed SSE more than three times within 6 months. However, SSE was often performed twice within the 6 months of follow-up in the intervention group, corresponding to the recommended interval (once every third month). All patients treated for melanoma are informed about SSE and encouraged to do so regularly. We do not believe in a specific “spill-over” effect from the intervention to standard care because the nurses performing the intervention did not take part in the physician-led follow-up of the control group and vice versa. The most sensitive sign of a new melanoma is a change in an existing mole [50], and changes might not be detected if SSE is performed too often. Furthermore, SSE was performed with higher confidence among patients in the intervention group compared to the control group. Overall, this highlights the feasibility of providing SSE education to this population.

### Health-related quality of life and patient activation

Through the MELACARE intervention, we aimed to empower patients to perform SSE and manage potential FCR. The intervention group did show an improvement in patient activation, which has not been assessed in the previous RCTs [27–30], and this may indicate that patients indeed felt more empowered.

We also found a positive effect of the intervention on HRQoL, consistent with previous studies evaluating interventions to reduce FCR [28, 30]. This supports the potential for FCR-focused interventions to impact HRQoL positively.

### Symptom assessment

The response rate of FACT-M was high before each of the three nurse sessions, and relevant actions were provided according to predefined guidelines. Together with previous studies, it supports the feasibility of using PROs in melanoma follow-up [51].

### Strengths and limitations

Study strengths include the low attrition rate during follow-up, the high completion rate of questionnaires at all follow-up times, and high adherence. Furthermore, as fidelity and interrater fidelity were high, we are confident that the intervention was delivered as planned. A key strength of the RCT design is its ability to minimise confounding and establish causality. However, the study has some limitations, which reduce the abovementioned advantages. We did not reach our target sample size of 170 participants per group, which reduces statistical power, increasing the risk of missing an actual effect and limiting the generalisability of our findings.

## Conclusion

The MELACARE intervention did not have a conclusive effect on improving psychosocial well-being in melanoma survivors. However, in the explorative analyses, the intervention group fared better regarding two secondary outcomes: HRQoL and patient activation. We cannot determine whether the lack of effect on our primary outcome, FCR, is due to the smaller-than-desired sample size (type II error) or because the intervention actually did not have an effect. Nonetheless, so far, we conclude that empowering patients with tools for systematic SSE and controlling FCR while abandoning regular clinical follow-up in a population of early-stage melanoma does not seem harmful to psychosocial symptoms at 6 months of follow-up. Further follow-up will hopefully show if this is also safe in the long term and with lower healthcare costs at 5-year follow-up. The high fidelity and adherence of this kind of intervention provide knowledge that can be used in planning future interventions or follow-up programs for early-stage melanoma.

## Supplementary Information

Below is the link to the electronic supplementary material.

Supplementary Material 1 (PDF 581 KB)

## Data Availability

By Danish legislation, sharing patient data with third parties is prohibited; therefore, the data is available exclusively, as presented in this paper. The authors declare no competing interests.
