# Supplementary material for: Employing skin self-examination and fear of cancer recurrence management in early-stage melanoma follow-up: evaluation of the MELACARE intervention in a randomised controlled trial
Source: J Cancer Surviv. 2025 Jun 12;20(Suppl 1):606–17. doi: 10.1007/s11764-025-01841-1 (PMC13676537; doi:10.1007/s11764-025-01841-1)
Supplement: Supplementary file 1 [file 11764_2025_1841_MOESM1_ESM.pdf]

Online Resource 1. The MELACARE intervention

At the nurse sessions, patients will be educated in skin self-examination (SSE) and given support to cope with fear of cancer recurrence (FCR). The melanoma nurses will address FCR and concerns related to performing SSE and assess symptoms using the Functional Assessment of Cancer Therapy-Melanoma (FACT-M) responses during each nurse session [31].

Education in SSE will include [31]:

- In-person training on SSE techniques,
- Information on the recommended frequency of performing SSE every three months,
- An educational booklet, and
- Guidance on recognising concerning symptoms using the ABCDE criteria.

The nurses will introduce the following techniques [31]:

- Detached Mindfulness (DM): Helps patients observe their thoughts and feelings without fixation or reaction, improving attention control and reducing the influence of worry on self-identity.
- Worry Postponement (WP): Encourages patients to record worries and postpone them to a set time, which helps challenge beliefs about uncontrollable worry and often makes revisiting these worries unnecessary.

Sessions Breakdown [31]**:**

Within six months of randomisation:

Nurse session 1

- **Content:**
    - Exploration of the patient's melanoma story
    - Assessment of FACT-M responses
    - Training of SSE skills and introduction to the ABCDE criteria
    - Provision of the patient information booklet
- **Duration:** 60 minutes
- **Mode:** In-person

Nurse session 2

- **Content:**
    - Follow-up from the previous session
    - Assessment of FACT-M responses
    - Exploration of emotions, thoughts, and behaviours related to melanoma, fear of cancer recurrence (FCR), and SSE
    - Attention to patient's coping strategies and introduction to DM and WP
    - Repetition of SSE skills and ABCDE criteria
- **Duration:** 60 minutes

- **Mode:** In-person or video consultation

Nurse session 3

- **Content:**
  - Follow-up from the last session, focusing on the use of DM and WP
  - Assessment of FACT-M responses
  - Repetition of SSE skills and the ABCDE criteria
- **Duration:** 30 minutes
- **Mode:** Telephone or video consultation

Physician consultation

- **Content:**
  - Evaluation of SSE skills
  - Demonstration on how to perform SSE
- **Duration:** 15 minutes
- **Mode:** In-person

At 12 months:

Session 4 (optional)

- **Content:**
  - Follow-up from the last session.
  - Assessment of FACT-M responses
  - Repetition of SSE skills and the ABCDE criteria
- **Duration:** 20 minutes
- **Mode:** Telephone consultation

At 24 months:

Session 5 (optional)

- **Content:**
  - Follow-up from the last session
  - Assessment of FACT-M responses
  - Repetition of SSE skills and the ABCDE criteria
- **Duration:** 20 minutes
- **Mode:** Telephone consultation

Online Resource 2. Nurse training program

The nurses who delivered the MELACARE intervention participated in a comprehensive 2-day training program to provide in-depth knowledge of skin self-examination, Detached Mindfulness, Worry Postponement, melanoma, and its management as outlined in the protocol [31]. Two psychologists and one medical doctor conducted the training.

Topics of the lessons (each 45 minutes) [31]:

- Study protocol (two lessons)
- Nurse manual (three lessons)
- Melanoma, detection of recurrence, and follow-up (two lessons)
- Skin self-examination (two lessons)
- Functional Assessment of Cancer Therapy-Melanoma and flowcharts for actions (two lessons)
- Fear of cancer recurrence (one lesson)
- Empathic listening (three lessons)
- Working with worries and use of Worry Postponement (three lessons)
- Detached Mindfulness practice (three lessons)

Online Resource 3. Predefined actions according to patients' Functional Assessment of Cancer Therapy-Melanoma questionnaire answers used at the nurse sessions

FACT-M item	Possible actions if participants replied "somewhat" or worse
GP 1	• Not melanoma-related; refer to the general practitioner if the symptom does not resolve within two weeks.<br>• Meets ICD-10 criteria; refer to the general practitioner.<br>• Does not meet ICD-10 criteria; refer to the general practitioner if the symptom does not resolve within two weeks.<br>• Lymphedema physiotherapy.<br>• Rehabilitation.<br>• Follow-up in the outpatient clinic if symptom does not resolve within two weeks.<br>• Follow-up in the outpatient clinic as soon as possible.<br>• Support through sessions.
GP 2	• Not melanoma-related; refer to the general practitioner if the symptom does not resolve within two weeks.<br>• Follow-up in the outpatient clinic if symptom does not resolve within two weeks.<br>• Follow-up in the outpatient clinic as soon as possible.<br>• Assistance through sessions.
GP 3	• Not melanoma-related; refer to the general practitioner if the symptom does not resolve within two weeks.<br>• Lymphedema physiotherapy.<br>• Rehabilitation.<br>• Follow-up in the outpatient clinic if symptom does not resolve within two weeks.<br>• Follow-up in the outpatient clinic as soon as possible.<br>• Support through sessions.
GP 4	• Not melanoma-related; refer to the general practitioner if the symptom does not resolve within two weeks.<br>• Lymphedema physiotherapy.<br>• Rehabilitation.<br>• Follow-up in the outpatient clinic if symptom does not resolve within two weeks.<br>• Follow-up in the outpatient clinic as soon as possible.<br>• Support through sessions.
GP 5	• Lymphedema physiotherapy.<br>• Rehabilitation.<br>• Follow-up in the outpatient clinic if symptom does not resolve within two weeks.<br>• Follow-up in the outpatient clinic as soon as possible.<br>• Support through sessions.
GP 6	• Not melanoma-related; refer to the general practitioner if the symptom does not resolve within two weeks.<br>• Lymphedema physiotherapy.<br>• Rehabilitation.<br>• Follow-up in the outpatient clinic if symptom does not resolve within two weeks.<br>• Follow-up in the outpatient clinic as soon as possible.<br>• Support through sessions.
GP 7	• Not melanoma-related; refer to the general practitioner if the symptom does not resolve within two weeks.<br>• Lymphedema physiotherapy.<br>• Rehabilitation.<br>• Follow-up in the outpatient clinic if symptom does not resolve within two weeks.<br>• Follow-up in the outpatient clinic as soon as possible.<br>• Support through sessions.
GS 1	• Not melanoma-related; refer to the general practitioner if the symptom does not resolve within two weeks.<br>• Support through sessions.
GS 2	• Not melanoma-related; refer to the general practitioner if the symptom does not resolve within two weeks.

	• Support through sessions.
GS 3	• Not melanoma-related; refer to the general practitioner if the symptom does not resolve within two weeks.<br>• Support through sessions.
GS 4	• Support through sessions.
GS 5	• Support through sessions.
GS 6	• Not melanoma-related; refer to the general practitioner if the symptom does not resolve within two weeks.<br>• Support through sessions.
GS 7	• Not melanoma-related; refer to the general practitioner if the symptom does not resolve within two weeks.<br>• Lymphedema physiotherapy.<br>• Rehabilitation.<br>• Follow-up in the outpatient clinic if symptom does not resolve within two weeks.<br>• Follow-up in the outpatient clinic as soon as possible.<br>• Support through sessions.
GE 1	• Not melanoma-related; refer to the general practitioner if the symptom does not resolve within two weeks.<br>• Meets ICD-10 criteria; refer to the general practitioner.<br>• Does not meet ICD-10 criteria; refer to the general practitioner if the symptom does not resolve within two weeks.<br>• Support through sessions.
GE 2	• Support through sessions.
GE 3	• Meets ICD-10 criteria; refer to the general practitioner.<br>• Does not meet ICD-10 criteria; refer to the general practitioner if the symptom does not resolve within two weeks.<br>• Support through sessions.
GE 4	• Not melanoma-related; refer to the general practitioner if the symptom does not resolve within two weeks.<br>• Support through sessions.
GE 5	• Not melanoma-related; refer to the general practitioner if the symptom does not resolve within two weeks.<br>• Support through sessions.
GE 6	• Support through sessions.
GF 1	• Lymphedema physiotherapy.<br>• Rehabilitation.<br>• Follow-up in the outpatient clinic if symptom does not resolve within two weeks.<br>• Follow-up in the outpatient clinic as soon as possible.<br>• Support through sessions.<br>• Not melanoma-related; refer to general practitioner or employer
GF 2	• Support through sessions.<br>• Not melanoma-related; refer to general practitioner or employer
GF 3	• Not melanoma-related; refer to the general practitioner if the symptom does not resolve within two weeks.<br>• Meets ICD-10 criteria; refer to the general practitioner.<br>• Does not meet ICD-10 criteria; refer to the general practitioner if the symptom does not resolve within two weeks.<br>• Lymphedema physiotherapy.<br>• Rehabilitation.<br>• Follow-up in the outpatient clinic if symptom does not resolve within two weeks.<br>• Follow-up in the outpatient clinic as soon as possible.<br>• Support through sessions.
GF 4	• Support through sessions.
GF 5	• Not melanoma-related; refer to the general practitioner if the symptom does not resolve within two weeks.<br>• Meets ICD-10 criteria; refer to the general practitioner.<br>• Does not meet ICD-10 criteria; refer to the general practitioner if the symptom does not resolve within two weeks.

	• Lymphedema physiotherapy.<br>• Rehabilitation.<br>• Follow-up in the outpatient clinic if symptom does not resolve within two weeks.<br>• Follow-up in the outpatient clinic as soon as possible.<br>• Support through sessions.
GF 6	• Meets ICD-10 criteria; refer to the general practitioner.<br>• Does not meet ICD-10 criteria; refer to the general practitioner if the symptom does not resolve within two weeks.<br>• Support through sessions.
GF 7	• Not melanoma-related; refer to the general practitioner if the symptom does not resolve within two weeks.<br>• Support through sessions.
M 1	• Lymphedema physiotherapy.<br>• Rehabilitation.<br>• Follow-up in the outpatient clinic if symptom does not resolve within two weeks.<br>• Follow-up in the outpatient clinic as soon as possible.<br>• Support through sessions.
M 2	• Follow-up in the outpatient clinic as soon as possible.<br>• Support through sessions.
M 3	• Follow-up in the outpatient clinic as soon as possible.<br>• Support through sessions.<br>• Follow-up in the outpatient clinic when convenient.
B 1	• Not melanoma-related; refer to the general practitioner if the symptom does not resolve within two weeks.<br>• Support through sessions.<br>• Not melanoma-related; refer to the general practitioner as soon as possible.<br>• The patient is known to have this symptom and is receiving treatment.
ITU 4	• Not melanoma-related; refer to the general practitioner if the symptom does not resolve within two weeks.<br>• Lymphedema physiotherapy.<br>• Rehabilitation.<br>• Follow-up in the outpatient clinic if symptom does not resolve within two weeks.<br>• Follow-up in the outpatient clinic as soon as possible.<br>• Support through sessions.<br>• The patient is known to have this symptom and is receiving treatment.
AN 10	• Not melanoma-related; refer to the general practitioner if the symptom does not resolve within two weeks.<br>• Support through sessions.<br>• Not melanoma-related; refer to the general practitioner as soon as possible.<br>• The patient is known to have this symptom and is receiving treatment.
HEP 3	• Not melanoma-related; refer to the general practitioner if the symptom does not resolve within two weeks.<br>• Support through sessions.<br>• Not melanoma-related; refer to the general practitioner as soon as possible.<br>• The patient is known to have this symptom and is receiving treatment.
C 1	• Not melanoma-related; refer to the general practitioner if the symptom does not resolve within two weeks.<br>• Support through sessions.<br>• Not melanoma-related; refer to the general practitioner as soon as possible.<br>• The patient is known to have this symptom and is receiving treatment.
C 6	• Not melanoma-related; refer to the general practitioner if the symptom does not resolve within two weeks.<br>• Meets ICD-10 criteria; refer to the general practitioner.<br>• Does not meet ICD-10 criteria; refer to the general practitioner if the symptom does not resolve within two weeks.<br>• Support through sessions.
M 5	• Not melanoma-related; refer to the general practitioner if the symptom does not resolve within two weeks.

	• Support through sessions.<br>• Not melanoma-related; refer to the general practitioner as soon as possible.<br>• The patient is known to have this symptom and is receiving treatment.
M 6	• Not melanoma-related; refer to the general practitioner if the symptom does not resolve within two weeks.<br>• Support through sessions.<br>• Not melanoma-related; refer to the general practitioner as soon as possible.<br>• The patient is known to have this symptom and is receiving treatment.
ITU 3	• Not melanoma-related; refer to the general practitioner if the symptom does not resolve within two weeks.<br>• Meets ICD-10 criteria; refer to the general practitioner.<br>• Does not meet ICD-10 criteria; refer to the general practitioner if the symptom does not resolve within two weeks.<br>• Lymphedema physiotherapy.<br>• Rehabilitation.<br>• Follow-up in the outpatient clinic if symptom does not resolve within two weeks.<br>• Follow-up in the outpatient clinic as soon as possible.<br>• Support through sessions.
MS 8	• Support through sessions.
M 8	• Support through sessions.
M 9	• Not melanoma-related; refer to the general practitioner if the symptom does not resolve within two weeks.<br>• Meets ICD-10 criteria; refer to the general practitioner.<br>• Does not meet ICD-10 criteria; refer to the general practitioner if the symptom does not resolve within two weeks.<br>• Support through sessions.
HI 7	• Not melanoma-related; refer to the general practitioner if the symptom does not resolve within two weeks.<br>• Meets ICD-10 criteria; refer to the general practitioner.<br>• Does not meet ICD-10 criteria; refer to the general practitioner if the symptom does not resolve within two weeks.<br>• Lymphedema physiotherapy.<br>• Rehabilitation.<br>• Follow-up in the outpatient clinic if symptom does not resolve within two weeks.<br>• Follow-up in the outpatient clinic as soon as possible.<br>• Support through sessions.
M 10	• Lymphedema physiotherapy.<br>• Follow-up in the outpatient clinic if symptom does not resolve within two weeks.<br>• Follow-up in the outpatient clinic as soon as possible.<br>• Support through sessions.
M 11	• Lymphedema physiotherapy.<br>• Follow-up in the outpatient clinic if symptom does not resolve within two weeks.<br>• Follow-up in the outpatient clinic as soon as possible.<br>• Support through sessions.
M 12	• Lymphedema physiotherapy.<br>• Follow-up in the outpatient clinic if symptom does not resolve within two weeks.<br>• Follow-up in the outpatient clinic as soon as possible.<br>• Support through sessions.
M 13	• Lymphedema physiotherapy.<br>• Follow-up in the outpatient clinic if symptom does not resolve within two weeks.<br>• Follow-up in the outpatient clinic as soon as possible.<br>• Support through sessions.
M 14	• Lymphedema physiotherapy.<br>• Follow-up in the outpatient clinic if symptom does not resolve within two weeks.<br>• Follow-up in the outpatient clinic as soon as possible.<br>• Support through sessions.
M 15	• Lymphedema physiotherapy.<br>• Follow-up in the outpatient clinic if symptom does not resolve within two weeks.

	• Follow-up in the outpatient clinic as soon as possible.<br>• Support through sessions.
M 16	• Support through sessions.
M 17	• Not melanoma-related; refer to the general practitioner if the symptom does not resolve within two weeks.<br>• Rehabilitation.<br>• Follow-up in the outpatient clinic if symptom does not resolve within two weeks.<br>• Follow-up in the outpatient clinic as soon as possible.<br>• Support through sessions.<br>• The patient is known to have this symptom and is receiving treatment.

ICD: International classification of diseases, FACT-M: Functional Assessment of Cancer Therapy-Melanoma

Online Resource 4. Study-specific questionnaire

<u>For participants in the intervention group:</u>

1. **Questions concerning skin self-examination**
   a. How many times have you thoroughly examined your skin as the nurse taught you in the last six months?
      - 0 times
      - one time
      - two times
      - three times
      - More than three times

   b. How confident are you in performing skin self-examination?
      - Not at all confident
      - Somewhat confident
      - Confident
      - Very confident

2. **Questions concerning the use of coping tools**
   a. How many times in the last six months have you used the tools for coping with the fear of recurrence of your melanoma, which you were taught by the nurse, such as Detached Mindfulness and Worry Postponement?
      - 0 times
      - 1-2 times
      - 3-4 times
      - 5-6 times
      - More than six times

   b. How helpful was Detached Mindfulness to you?
      - Not at all helpful
      - Somewhat helpful
      - Helpful
      - Very helpful

   c. How helpful was Worry Postponement to you?
      - Not at all helpful
      - Somewhat helpful
      - Helpful
      - Very helpful

3. **Questions concerning the booklet "Melanoma – Questions and Answers"**
   a. How much of the "Melanoma – Questions and Answers" booklet have you read?
      - Did not read it
      - Read under half
      - Read over half

- Read it all

b. How many times have you used the booklet "Melanoma – Questions and Answers" in the last six months?
    - 0 times
    - 1-2 times
    - 3-4 times
    - 5-6 times
    - More than six times

4. Questions concerning satisfaction and benefits of the follow-up program

a. Overall, how satisfied are you with the follow-up care after treatment for melanoma?
    - Not at all satisfied
    - Somewhat satisfied
    - Satisfied
    - Very satisfied

b.

**By participating in this follow-up, I have learned:**				
	Strongly disagree	Disagree	Agree	Strongly agree
How often I should do skin self-examination				
How to perform skin self-examination				
To talk more openly about my melanoma with my family and friends				
To cope better with my fear of my melanoma will recur or a new melanoma				
To use Detached Mindfulness and/or Worry Postponement				
Where to find more information about support for me or my family				

5. Questions concerning practical matters regarding your visits to the outpatient clinic

a. Which mode of transportation do you most often use when attending an appointment at the outpatient clinic?
    - Private car
    - Public transportation
    - Taxi
    - Transportation services (both paid by yourself or the hospital)
    - Bicycle
    - Walking
    - Other:______________

b. How many minutes does it usually take you to get to the outpatient clinic, including time spent finding a parking space and walking to/from the bus stop or train station? Please provide an approximate number of minutes:______________

c. How long is your journey in kilometres approximately? Please provide an approximate number of kilometres:_______________

For participants in the control group:

1. **Questions concerning skin self-examination**
   a. How many times have you thoroughly examined your skin for changes or new moles in the last six months?
      - 0 times
      - one time
      - two times
      - three times
      - More than three times

   b. How confident are you in performing skin self-examination?
      - Not at all confident
      - Somewhat confident
      - Confident
      - Very confident

2. **Questions concerning satisfaction with follow-up program**
   a. Overall, how satisfied are you with the follow-up care after treatment for melanoma?
      - Not at all satisfied
      - Somewhat satisfied
      - Satisfied
      - Very satisfied

3. **Questions concerning practical matters regarding your visits to the outpatient clinic**
   a. Which mode of transportation do you most often use when attending an appointment at the outpatient clinic?
      - Private car
      - Public transportation
      - Taxi
      - Transportation services (both paid by yourself or the hospital)
      - Bicycle
      - Walking
      - Other:_______________

   b. How many minutes does it usually take you to get to the outpatient clinic, including time spent finding a parking space and walking to/from the bus stop or train station? Please provide an approximate number of minutes:_______________

   c. How long is your journey in kilometres approximately? Please provide an approximate number of kilometres:_______________

Online Resource 5. Targeted maximum likelihood estimation analyses

Targeted maximum likelihood estimation (TMLE) requires specifying a set of suitable machine learning algorithms for the outcome, the propensity score, and the missingness indicator. Table S1 shows the specifications for each analysis, i.e., the learning algorithms used in the ensembles for each analysis and the adjustment set of covariates for each analysis. The continuous outcomes were scaled to lie between 0 and 1 by dividing by (max-min) before applying TMLE. Afterwards, the results were transformed back to the original scale[1].

	Ensemble	Covariates[4]
Outcome	Generalised linear model[2], elastic net penalised regression, Bayesian additive regression trees model	Intervention status, outcome at baseline, age, sex, level of education, employment status, civil status, any children, stage
Propensity score	Elastic net penalised regression, Bayesian additive regression trees model	Outcome at baseline, age, sex, level of education, employment status, civil status, any children, stage
Missingness indicator	Elastic net penalized regression[3], Bayesian additive regression trees model	Intervention status, outcome at baseline, age, sex, level of education, employment status, civil status, any children, stage

[1]Reference: Frank HA, Karim ME. Implementing TMLE in the presence of a continuous outcome. Research Methods in Medicine & Health Sciences. 2023;5(1):8-19. doi:10.1177/26320843231176662

[2]Generalised linear models were not included in the ensemble for HRQoL, due to separation in the logistic regression model for outcome.

[3]Elastic net penalised regression was not included in the subgroup analyses for men and FCR ≤ 11 due to too few observations with missing FCR at 6 months.

[4]A covariate is not included if it is part of the subgroup analysis, e.g., sex is not adjusted for in the subgroup analyses for sex.

Online Resource 6. Additional analyses

We also performed the analyses as proposed in the protocol. Linear mixed models were used to estimate the effect on the primary and secondary outcomes at 6 months. The models were constrained at baseline, so the intervention and control groups had the same intercept. A random intercept was included for each individual. The models were adjusted for stage since randomisation was stratified by clinical stage. The model assumptions, e.g. about normal residuals, were, however, questionable for all outcomes, some more than others. The protocol also proposed an alternative method where the outcomes would be normalised[1]. However, this approach would require transformation from the normalised scale back to the original scale, which would make the interpretation challenging partly because the marginal interpretation would be lost.

Mean differences in fear of cancer recurrence, distress, anxiety, depression, workability, patient activation, and health-related quality of life between intervention and control groups at six months of follow-up using linear mixed models

**Outcome**	**Between-group difference**	
	Estimate (95%CI)	p-value
	Mean difference	
Fear of cancer recurrence	-0.86 (-3.4;1.68)	0.51
Distress	-0.26 (-1;0.45)	0.48
Anxiety	-0.6 (-1.5;0.3)	0.19
Depression	-0.73 (-1.7;0.24)	0.14
Workability	1.33 (-0.89;3.54)	0.24
Patient activation	2.37 (1.3;4.32)	0.005
Health status[2]	2.74 (1.4;5.24)	0.002

SD: standard deviation, CI: confidence interval

[1]*Reference: Proust C, Jacqmin-Gadda H, Taylor JMG, Ganiayre J, Commenges D. A nonlinear model with latent process for cognitive evolution using multivariate longitudinal data. Biometrics. 2006;62:1014–24. doi:10.1111/j.1541-0420.2006.00573.x*

[2]*Health status is related to the health-related quality of life measure EQ-5D-3L, where a health status of 1 indicates "perfect health.*

Online Resource 7. Subgroup analysis of the intervention effect within subgroups, including base-line FCR levels, age, gender, and civil status, on fear of cancer recurrence at six-month follow-up

**Subgroup**	**Estimate (95%CI)[1]**	**p-value**
Fear of cancer recurrence at baseline		
> 11	0.77 (-3.86;5.42)	0.74
≤ 11	-0.57 (-2.09;0.95)	0.46
Age		
≥ 60	1.1 (-1.89;4.08)	0.47
< 60	-4.41 (-9;0.18)	0.06
Gender		
Women	-3.15 (-7.12;0.82)	0.12
Men	-1.06 (-3.97;1.85)	0.47
Civil status		
Married/cohabitating	-0.78 (-3.55;1.99)	0.58
Separated/divorced/never married/widowed	-4.08 (-10.1;1.94)	0.18

CI: confidence interval

[1]*Targeted maximum likelihood estimation (TMLE) was used to estimate the intervention effect at six months follow-up.*

Online Resource 8. Referrals/advice/support made by melanoma nurses among 69 intervention group patients during Sessions 1, 2, and 3

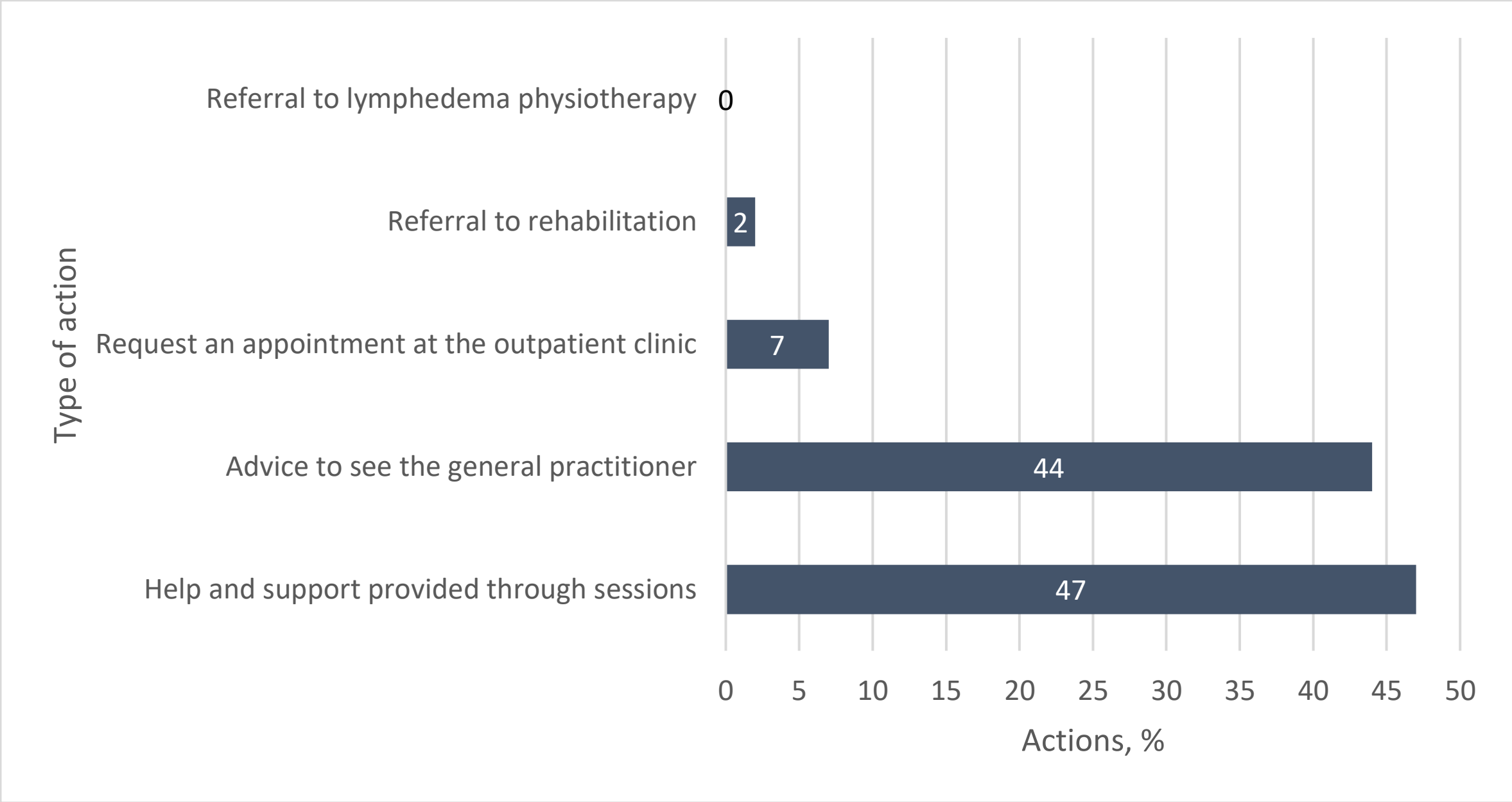

Online Resource 9. Self-reported use and usefulness of intervention components and skills learnt among 69 patients in the intervention group at six months of follow-up

**Outcome**					
	0 times	1-2 times	3-4 times	5-6 times	>6 times
Use of coping tools, N (%)	34 (50)	23 (34)	8 (12)	1 (1)	2 (3)
Use of booklet, N (%)	23 (34)	37 (54)	5 (8)	2 (3)	1 (1)
	Did not read it	Read under half	Read over half	Read it all	
Read the booklet, N (%)	3 (4)	12 (18)	8 (12)	45 (66)	
	Not at all useful	Partly useful	Useful	Very useful	
Usefulness of					
Detached mindfulness, N (%)	30 (44)	21 (31)	13 (19)	4 (6)	
Worry postponement, N (%)	27 (40)	23 (34)	15 (22)	3 (4)	
	Completely disagree	Disagree	Agree	Completely agree	
Learned how often to do SSE, N (%)	0 (0)	0 (0)	29 (43)	39 (57)	
Learned how to do SSE, N (%)	0 (0)	1 (1)	31 (46)	36 (53)	
Learned to talk about the melanoma diagnosis, N (%)	1 (1)	3 (4)	30 (44)	34 (50)	
Learned to better cope with the melanoma diagnosis, N (%)	0 (0)	4 (6)	38 (56)	26 (38)	
Learned how to use coping strategies, N (%)	6 (9)	10 (15)	37 (54)	15 (22)	
Learned where to find more information and support, N (%)	1 (1)	2 (3)	39 (58)	26 (38)	

SSE: skin self-examination
